# Developing global goals for soil health research and action: report on the “Soil Stars Heligan Summit,” The Lost Gardens of Heligan, UK, March 2026

**DOI:** 10.1093/sumbio/qvag038

**Published:** 2026-09-12

**Authors:** Jo Handelsman, Bridget Emmett, Chris Schadt, Ian R Sanders, David Evans, Gwyn A Beattie, Jennifer B H Martiny, Thomas Crowther, Joana Falcão Salles, Mette Haubjerg Nicolaisen, Lucy Harper, Mark Stevenson, Anna Edlund, Peter Byck, Matthew B Sullivan, Bill McGlashan, Meredith Lischer, Kirsten Benjamin, Jane Franch, Martin Voss, Paul Hawken, Jens Hartmann, Ginger Krieg Dosier, Veronica Garcia, Michael Sauer, Max M Häggblom, Evelyn Doyle, Tim Smit, Dan Ryan, Michael Depledge, Philip Marsden, Janet K Jansson, Jack A Gilbert

**Affiliations:** The Soil Stars, Applied Microbiology International, Cambridge CB1 2LA, United Kingdom; Wisconsin Institute for Discovery and Department of Plant Pathology, University of Wisconsin–Madison, Madison, WI 53706, United States; The Soil Stars, Applied Microbiology International, Cambridge CB1 2LA, United Kingdom; Soils and Land Use, UK Centre for Ecology & Hydrology, Bangor, LL57 2UW, United Kingdom; The Soil Stars, Applied Microbiology International, Cambridge CB1 2LA, United Kingdom; Biosciences Division, Oak Ridge National Laboratory, Oak Ridge, TN 37831, United States; The Soil Stars, Applied Microbiology International, Cambridge CB1 2LA, United Kingdom; Department of Ecology and Evolution, University of Lausanne, Lausanne, CH-1015, Switzerland; The Soil Stars, Applied Microbiology International, Cambridge CB1 2LA, United Kingdom; Oath Biome, San Francisco, CA 94129, United States; The Soil Stars, Applied Microbiology International, Cambridge CB1 2LA, United Kingdom; Department of Plant Pathology, Entomology and Microbiology, Iowa State University, Ames, IA 50011, United States; The Soil Stars, Applied Microbiology International, Cambridge CB1 2LA, United Kingdom; Department of Ecology and Evolutionary Biology, University of California, Irvine, Irvine, CA 92697, United States; The Soil Stars, Applied Microbiology International, Cambridge CB1 2LA, United Kingdom; Oath Biome, San Francisco, CA 94129, United States; BRANCH Institute, Zug 6300, Switzerland; King Abdullah University of Science and Technology (KAUST), Thuwal 23955-6900, Saudi Arabia; The Soil Stars, Applied Microbiology International, Cambridge CB1 2LA, United Kingdom; Groningen Institute for Evolutionary Life Sciences, University of Groningen, Groningen 9712 CP, Netherlands; The Soil Stars, Applied Microbiology International, Cambridge CB1 2LA, United Kingdom; Department of Plant and Environmental Sciences, University of Copenhagen, Frederiksberg, 1165 Copenhagen K, Denmark; The Soil Stars, Applied Microbiology International, Cambridge CB1 2LA, United Kingdom; The Soil Stars, Applied Microbiology International, Cambridge CB1 2LA, United Kingdom; Oath Biome, San Francisco, CA 94129, United States; The Soil Stars, Applied Microbiology International, Cambridge CB1 2LA, United Kingdom; Oath Biome, San Francisco, CA 94129, United States; The Soil Stars, Applied Microbiology International, Cambridge CB1 2LA, United Kingdom; School of Sustainability, Arizona State University, Tempe, AZ 85281, United States; Walter Cronkite School of Journalism and Mass Communication, Arizona State University, Phoenix, AZ 85004, United States; The Soil Stars, Applied Microbiology International, Cambridge CB1 2LA, United Kingdom; Department of Microbiology and Center of Microbiome Science, The Ohio State University, Columbus, OH 43210, United States; Oath Biome, San Francisco, CA 94129, United States; Oath Biome, San Francisco, CA 94129, United States; Oath Biome, San Francisco, CA 94129, United States; Oath Biome, San Francisco, CA 94129, United States; Oath Biome, San Francisco, CA 94129, United States; Project Drawdown, St. Paul, MN 55101, United States; Oath Biome, San Francisco, CA 94129, United States; BIOME Consortia, United States; American Society for Microbiology, Washington, DC 20036, United States; Federation of European Microbiological Societies (FEMS), Delft, 2600 BA, Netherlands; OMV AG, Department of Biotechnology, Vienna 1190, Austria; Federation of European Microbiological Societies (FEMS), Delft, 2600 BA, Netherlands; Department of Biochemistry and Microbiology, Rutgers University, New Brunswick, NJ 08901, United States; Federation of European Microbiological Societies (FEMS), Delft, 2600 BA, Netherlands; School of Biology and Environmental Science, University College Dublin, Belfield, D04 V1W8, Dublin 4, Ireland; The Soil Stars, Applied Microbiology International, Cambridge CB1 2LA, United Kingdom; The Lost Gardens of Heligan, Pentewan, St. Austell, Cornwall, PL26 6EN, United Kingdom; Eden Project, Bodelva, St Austell, Cornwall, PL24 2SG, United Kingdom; The Soil Stars, Applied Microbiology International, Cambridge CB1 2LA, United Kingdom; The Lost Gardens of Heligan, Pentewan, St. Austell, Cornwall, PL26 6EN, United Kingdom; Eden Project, Bodelva, St Austell, Cornwall, PL24 2SG, United Kingdom; The Lost Gardens of Heligan, Pentewan, St. Austell, Cornwall, PL26 6EN, United Kingdom; Eden Project, Bodelva, St Austell, Cornwall, PL24 2SG, United Kingdom; European Centre for Environment and Human Health, University of Exeter Medical School, Penryn Campus, Cornwall TR10 9FE, United Kingdom; Animal and Plant Health Agency (APHA), Weybridge: Woodham Lane, New Haw, Addlestone, Surrey, KT15 3NB, United Kingdom; Royal Society of Literature, Somerset House, Strand, London WC2R 1LA, United Kingdom; The Soil Stars, Applied Microbiology International, Cambridge CB1 2LA, United Kingdom; Oath Biome, San Francisco, CA 94129, United States; Pacific Northwest National Laboratory, Richland, WA 99354, United States; The Soil Stars, Applied Microbiology International, Cambridge CB1 2LA, United Kingdom; Oath Biome, San Francisco, CA 94129, United States; Department of Pediatrics, University of California San Diego, La Jolla, CA 92093, United States; Soil Health Center, Scripps Institution of Oceanography, University of California San Diego, La Jolla, CA 92093, United States

**Keywords:** soil health, microbial inoculants, biological fertilizers, regenerative agriculture, XPRIZE, soil carbon, mission-led innovation, soil microbiome

## Abstract

Soil underpins food production, freshwater, carbon storage and biodiversity, yet over 40% of the world’s soils are now degraded, eroding at rates up to 1000 times their formation rate. Microbial technologies have matured and can now aid soil recovery, but deployment has lagged due to absent shared targets, harmonized metrics, and coherent reward structures to mobilize solvers across academia, industry, philanthropy, and government. Building on major initiatives (e.g. A Soil Deal for Europe, the FAO Global Soil Partnership, etc.) a consensus has emerged on the need for actionable, transformative advances in soil health. To translate that consensus into coordinated action, The Soil Stars, a multidisciplinary coalition of scientists, communicators, investors, policymakers, and farming-systems leaders, convened the inaugural Soil Stars Heligan Summit in March 2026 at The Lost Gardens of Heligan, Cornwall, United Kingdom, with a public launch at the Eden Project’s Anthropy 2026 forum. The summit combined a grand-missions hack-a-thon with roundtables on the climate-society interface, the soil carbon-fertility-microbiome nexus, and deployment hurdles. The primary outcome is the first draft of the Heligan Grand Challenges; seven XPRIZE-inspired challenges with concrete performance thresholds achievable within 24–36 months, alongside a path toward a formal Grand Challenges paper and five-year implementation roadmap.

**Sustainability statement**

The primary Sustainable Development Goal addressed by the Heligan Grand Challenges is SDG 2 (Zero Hunger). Healthy, productive soils are fundamental to food security, and the draft Challenges seek scalable approaches to restore degraded agricultural soils, reduce erosion and reliance on synthetic fertilizers, and maintain crop yields through improved biological function. By defining measurable targets and validation approaches, the initiative aims to accelerate the field evaluation of soil-management strategies capable of supporting resilient food production. The work may also contribute to SDG 6 (Clean Water and Sanitation), SDG 13 (Climate Action), SDG 15 (Life on Land), and SDG 17 (Partnerships for the Goals).

## Introduction

Soil is the thin biological skin between the Earth’s bedrock and atmosphere. Its contributions to the planet cannot be overstated as soil is the foundation on which the terrestrial food system, freshwater supply, terrestrial carbon stock, and most aboveground biodiversity depend. However, both degradation of soil quality and outright soil loss, or erosion, threaten the health of the planet and those who inhabit it. The Food and Agriculture Organization (FAO) estimates that 1.7 billion people live in areas where crop yields are declining due to soil degradation and that >40% of the world’s soils are now degraded (FAO 2015). The United Nations Convention to Combat Desertification (UNCCD) estimates that up to 40% of arable land is degraded with 100 million hectares of productive land lost each year (United Nations Convention to Combat Desertification 2022). Soils in many regions of the world are eroding at rates 10−1000 times the rate at which they form (Borrelli et al. 2021). In some of the most productive agricultural landscapes the consequences are already starkly visible (Thaler et al. 2021). For example, in the midwestern Corn Belt of the United States, one third of cropland has lost all of its A-horizon, the topmost soil layer that is rich with life and nutrients; loss of this layer is associated with a 6% reduction in crop yield (Thaler et al. 2021). Loss of soil structure releases carbon, contaminates groundwater, and degrades the soil’s microbial communities, which include the bacteria, fungi, archaea, protists, and viruses that drive nearly every ecosystem service that soils provide. Moreover, with nearly a third of the global land already experiencing moderate to high levels of soil erosion and degradation, further losses of agriculturally productive soils could have devastating effects on global food security (FAO 2015).

Another crisis facing the world’s agricultural system is the increasing reliance on chemical agriculture (pesticides and fertilizers) and industrial farming that prioritizes yield over sustainability. The Green Revolution in the latter half of the 20th century was responsible for impressive boosts in agricultural productivity driven by application of synthetic fertilizers and pesticides resulting in yield gains over the short term. Currently, those yield gains have plateaued and are starting to decline in many regions of the world (Ray et al. 2012). The dependence on chemical fertilizers recently became urgent again in 2026, when the outbreak of the war in Iran and the resulting closure of the Strait of Hormuz disrupted roughly one-third of global seaborne fertilizer exports, sharply raising fertilizer prices worldwide (Arita and Wang 2026). As a result, farmer profits are declining due to their reliance on increasingly expensive chemical treatments and equipment. A downstream impact of chemical agriculture and vast planting of monocultures (mainly soy and corn) is the loss of biodiversity, including birds and insects, that are keystone indicators of productive ecosystems. Life belowground is also negatively impacted with declines in microorganisms that support the soil food web and cycle carbon and other nutrients (Sauer et al. 2013, Lark et al. 2015, Schulte et al. 2017).

The scientific community has responded with an increasingly urgent and coordinated call to action. These include the EU Soil Mission and the Global Soil Partnership as well as more community-based calls for actions such as the Save Soil Foundation. Recently, it was argued that the microbiome must be harnessed to prevent global biodiversity loss (Peixoto RS et al. 2022) and subsequently that microbial solutions must be deployed against climate catastrophe (Peixoto R. et al. 2024). Microorganisms and microbial technologies can contribute to the UN Sustainable Development Goals, although challenges remain (Averill et al. 2022, Jansson and Wu 2023, Crowther et al. 2024). Companion movements have launched the IUCN Microbial Conservation Specialist Group as a global safeguard for microbial biodiversity (Gilbert et al. 2025, Peixoto Raquel et al. 2025). In parallel, microbial inoculants and beneficial microbes have been shown to deliver measurable real-world benefits across coral reefs (Delgadillo-Ordoñez et al. 2024), forest soils (Averill et al. 2022), and agricultural systems (Hijri 2016, Ulzen et al. 2016, Lutz et al. 2023, Wei et al. 2023, Francioli et al. 2025). The benefits of microbial consortia for crops has a long history, with studies demonstrating significant yield enhancements, disease suppression, and plant dry mass accumulation In diverse field settings (Raupach and Kloepper 1998, Jetiyanon et al. 2003). A recent field study of several crop species indicates that multistrain microbial biostimulants can improve crop growth and stress resilience under both conventional and reduced-input management (Francioli et al. 2025). Beyond conventional inoculants, emerging microbiome-modulation strategies include function-oriented microbial consortia, phages, microbiome transplantation and breeding, and synbiotics (Marian et al. 2026).

Yet scientific possibility has outpaced the deployment. There are no universally agreed upon metrics of soil biological function; validation challenges differ between laboratories, regions, and crop systems; the regulatory pathways governing microbial products were not designed for biological inputs; and farmers, regulators, and investors lack a shared language for what counts as success. The result is a paradox in which the most credible biological solutions exist as scattered demonstrations rather than as a deployable platform (Alzate Zuluaga et al. 2024). Closing that gap requires a deliberate shift from incremental, project-by-project research toward mission-led, outcome-defined goals to which solvers across sectors can converge. One model for doing this is the XPRIZE tradition of defining what must be achieved but not putting restrictions on how to achieve it (Diamandis and Kotler 2014). The Soil Stars Heligan Summit was convened to drive that shift in soil management using microbiome technologies or surveillance.

### Formation and governance of the Soil Stars

The Soil Stars grew out of a February 2024 workshop funded by Oath Inc., the parent company of Oath Biome, a public benefit company dedicated to microbial products to improve soil health, and initially convened to inform formation of the company’s Scientific Advisory Board (Beattie et al. 2024). Participants identified broader questions concerning microbial technologies and soil health and subsequently established the Soil Stars as a separate Research Network hosted by Applied Microbiology International (AMI) (https://appliedmicrobiology.org/advocacy-policy/research-networks.html). The title of the group was proposed by a teenage student who commented that there are rock stars, so why are not there soil stars? She fully appreciated the relationship between rocks and soil, and the group was taken with her humorous and scientifically astute suggestion so it adopted Soil Stars as the name. Although some membership overlaps with the Oath Biome Scientific Advisory Board, the Soil Stars is independently governed and includes representatives from the wider community, including other companies. The network has no dedicated operating budget; AMI provides in-kind administrative support. An Executive Committee meets monthly, with quarterly meetings of the wider membership.

Following this initial workshop, the Soil Stars developed organically as a diverse network of people committed to accelerating action on the global soil crisis. Many members had previously pursued this goal through research, public writing and speaking, policy development, and teaching, but shared the view that progress remained far too slow. The initial network brought together soil scientists, microbiologists, communicators, artists, entrepreneurs, investors, and policy actors to encourage lateral thinking and unconventional ideas. The Heligan meeting was conceived to translate this breadth of perspectives into visionary but practical solutions with the potential for substantial impact, using an XPRIZE-like incentive model to accelerate implementation.

### The meeting

The Soil Stars Heligan Summit was hosted and funded by AMI, with additional in-kind support from the American Society for Microbiology (ASM), The Federation of European Microbiology Societies (FEMS), BIOME Consortia, and Oath Biome. Participants were recruited through an open call to the Soil Stars membership, with AMI supporting members who were available to attend. ASM, FEMS, BIOME Consortia, the Eden Project, and Oath Biome each nominated and supported their own delegates; other companies and entities were invited to attend but declined. As the first in-person Soil Stars meeting, participation was limited to these groups and was not intended to constitute representative sampling of the wider soil-science or farming communities; the resulting Challenges are therefore presented as an initial draft for broader consultation. Farmer representation at this first Summit was limited and did not constitute systematic consultation with the wider farming community. Farmer and rancher perspectives entered principally through scientists who work directly with growers, film makers’ accounts of collaborative research conducted on working farms and ranches, and through discussions of adoption, cost and operational feasibility. The summit was deliberately compact with approximately thirty participants, two and a half days, and an explicit design intent to move from problem diagnosis to a concrete drafting output by the end of Day 2. The meeting venues were chosen to help contextualize what is at risk and what can be done. The Lost Gardens of Heligan is a 200-acre restored estate that itself is a story about reviving a degraded landscape, and the Eden Project is the world’s largest indoor biodiversity-themed exhibit and the host of the Anthropy 2026 forum.

### Day 1—grand missions and lightning roundtables

The first day opened with a welcome from Janet Jansson (Chair of The Soil Stars), who framed the meeting’s purpose and reviewed the agenda. Jo Handelsman then opened the “grand missions for soil science” hack-a-thon together with Jack Gilbert, Mark Stevenson, and Tom Crowther. Handelsman set out the scale of the soil erosion crisis, drawing on her work on soil biology and policy action for soils (Handelsman 2021). She highlighted Iowa, the most productive maize-growing region in the world, which loses on average around five tons of soil per acre per year while generating new soil at approximately half a ton per acre per year, so that soils are eroding at ~100 times the rate at which they are formed (O’Hara and Benson 2019, Thaler et al. 2021). These rates are typical of many regions of Africa, Asia, the Middle East, and others (Dargie et al. 2017). Crowther presented the framing connections between humans and the Earth that underpinned much of the meeting’s communication thread (Crowther et al. 2024).

The lightning roundtables alternated with continued drafting of grand missions. The first, “Global climate change coalition across societies,” included representatives from different international microbiology societies. It was chaired by Lucy Harper (AMI), with participation from Joana Falcão Salles (International Society of Microbial Ecology, ISME), Veronica Garcia (ASM), and Max Häggblom (FEMS) to consider how international microbiology societies could coordinate around a shared soil-and-climate agenda (Lennon et al. 2025). The discussion identified that, despite the depth of microbial expertise across these societies, there has been no shared mission-led platform at which their members could converge, which was identified as a gap that the Heligan Grand Challenges could later be explicitly designed to fill.

The second roundtable, “Interplay between soil carbon, fertility and microbes, a precious but poorly understood global resource,” chaired by Bridget Emmett with Mark Stevenson, Matt Sullivan, and Anna Edlund, explored the global heterogeneity of soil carbon dynamics. Emmett described the case she had led for the European Union’s Mission: A Soil Deal for Europe, on an analysis of soil monitoring data that indicated between 60% and 70% of European soils were unhealthy helped to unlock approximately one billion euros of EU investment and committed Member States to 100 living labs and lighthouses by 2027 (Veerman et al. 2020). This statistic was later confirmed by the Joint Research Centre’s nineteen-indicator EU Soil Observatory Dashboard launched in March 2023 indicating 62% of EU soils were unhealthy. The remaining panelists shared anecdotes across science and policy where behaviors are changing in response to growing awareness of soil health’s importance, as well as science examples emerging that increasingly resolve the mechanistic details of how microbes improve soil health.

The day closed with a tour of Heligan Gardens led by Sir Tim Smit, the estate’s co-founder, and an evening conversation with the documentary filmmaker Peter Byck and Mark Stevenson under the title “How to make soil sexy.” Using an interview style, Stevenson asked Byck about his experience using film and documentaries to convey important themes. Byck highlighted recent research projects in collaboration with farmers and ranchers in the United Sates that compared regenerative agricultural practices (e.g. rotational grazing) to conventional practices, highlighting the economic and environmental advantages of the former (https://rootssodeep.org/). The conversation emphasized communication as one component of implementation. Participants recognized strong evidence for the importance of soil microbiology to natural habitats and agroecosystem function, while acknowledging that the effectiveness and reproducibility of microbial interventions under field conditions remain uncertain and context dependent. Narrative, attention and trust were therefore discussed as barriers alongside continued scientific and translational work.

### Day 2—from planned agenda to direct drafting of challenges

Three substantive scientific threads dominated the first draft of the Heligan Grand Challenges. First was the central question of whether biological interventions can be made to perform consistently in real soils. Anna Edlund and Joana Falcão Salles argued that the field has demonstrated efficacy in principle and that the remaining questions are around consistent performance in the field, making microbial products survive formulation, storage, transport, and application, and the scientific understanding of mechanism in soils populated by competitive resident communities. Jennifer Martiny and Jens Hartmann stressed the importance of ecological and biogeochemical context, i.e. that the same inoculant can succeed in one soil and fail in another, and that this is now a tractable scientific question to answer mechanistically rather than simply left unsolved as a generic disclaimer. Gwyn Beattie with Kirsten Benjamin, Mette Nicolaisen, Chris Schadt, and Ian Sanders focused on a second thread, that of exploiting plant–microbe interactions. Sanders’s long-running work on arbuscular mycorrhizal fungal inoculants in tropical and African smallholder systems was highlighted as a demonstration that single-organism microbial inputs can produce large, reproducible field effects when matched correctly to crop and soil context (Ceballos et al. 2013, Rodriguez and Sanders 2015). The third example offered by Matt Sullivan with methane suppression in thawing permafrost. Sullivan’s group had shown that a plant polyphenol suppressed methane emission by >90%, but not by suppressing the methanogenic archaea themselves; instead, genome-resolved multiomics analyses revealed that the intervention caused a secondary microbial bloom, induced a native prophage in the dominant primary responder, and lysed that responder to feed 27 other microbial lineages that outcompeted the methanogens for hydrogen (McGivern et al. 2021, Riddell et al. 2025). The example was deployed to make a more general point, that AI-enabled multiomics now makes it possible both to design microbial interventions a priori and to detect unintended consequences (such as compensatory nitrous oxide production) before deployment (Gilbert and Zengler 2025).

The afternoon was spent in working-group drafting, building the Grand Challenge portfolio summarized in Fig. 1 and Table 1. The day closed with the evening event at Heligan Gardens that was open to 200 registered public attendees “The ground beneath our feet,” a conversation between The Edge (David Evans), Dan Ryan and Tom Crowther, with Mark Stevenson moderating. That session focused on how the Challenges could connect to a wider cultural and civic narrative, and it set up the public launch on Day 3.

**Figure 1:**
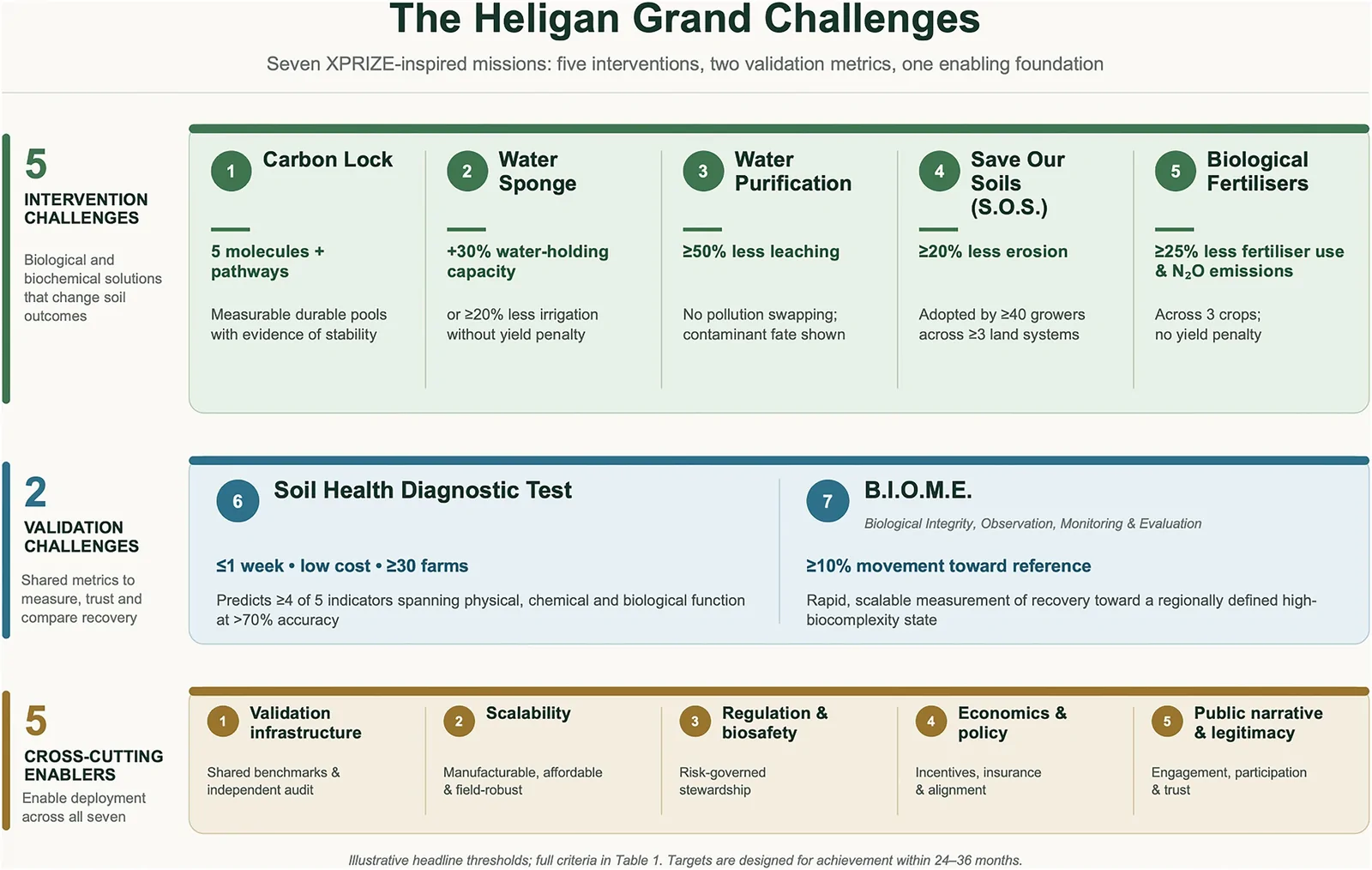
The Heligan Grand Challenges. The portfolio for seven grand challenges comprises five Intervention Challenges (green), which specify biological or biochemical solutions to improve a facet of soil health, and two Validation Challenges (blue), which define how those solutions are measured, trusted, and compared. Each challenge is shown with its illustrative headline threshold (full criteria in Table 1): Carbon Lock (durable, slow-turnover soil-carbon pools), Water Sponge (+30% water-holding capacity or − 20% irrigation demand), Water Purification (≥50% reduction in contaminant leaching with no pollution swapping), Save Our Soils/S.O.S. (≥20% reduction in erosion across ≥ 40 growers), Biological Fertilizers (≥25% reduction in synthetic fertilizer use and N₂O emissions with no yield penalty), the Soil Health Diagnostic Test (a rapid “soil blood test” predicting ≥ 4 of 5 indicators at >70% accuracy), and B.I.O.M.E. (≥10% movement toward a high-biocomplexity reference state). All seven rest on five cross-cutting enablers (tan)—validation infrastructure, scalability, regulation and biosafety, economics and policy, and public legitimacy—that operate across every challenge and must be addressed for any solution to be deployed at scale. Targets are illustrative thresholds achievable within 24–36 months; N₂O, nitrous oxide.

**Table 1:** The Heligan Challenges.

Challenge	Core mission	Illustrative winning threshold	Category	Why it matters
**(i) Carbon Lock Challenge**	Drive plant- and microbially-derived soil carbon into durable, slow-turnover pools that are not sensitive to rapid mineralization.	Identify five carbon-lock molecules and the pathways to increase them; demonstrate measurable increases in the corresponding soil pools over 24 months, with credible evidence of stability within 36 months and predicted stability over decades to centuries.	**Intervention**	Links microbiology directly to climate mitigation and to the MRV infrastructure required for credible soil-carbon credits.
**(ii) Water Sponge Challenge**	Increase the water-holding capacity of degraded and desiccated soils through biological restoration of soil structure.	Achieve a 30% increase in soil water-holding capacity and/or a ≥ 20% reduction in irrigation demand, with no yield or environmental penalty, across ≥ 3 soil types and climate regions. Within 24 months.	**Intervention**	Provides robust, transferable strategies to improve water retention in water-impoverished soils, supporting plant growth and soil sustainability.
**(iii) Water Purification Challenge**	Restore soil biology to transform or stabilise contaminants (nitrate, phosphate, or a degradable agrochemical) into benign end-states—not by passive retention alone—protecting both soils and water.	≥50% reduction in leaching of a target contaminant into streams and groundwater across ≥ 3 soil types and climate regions, within 24 months, subject to three guardrails: (i) No pollution swapping—no net increase in GHG emissions (CO₂e), with N₂O measured explicitly; (ii) no soil-health penalty—no accumulation of the parent contaminant or toxic metabolites; (iii) demonstrated fate—a contaminant mass-balance showing genuine transformation rather than temporary sorption.	**Intervention**	Connects soil recovery to water security and public health, addressing contaminant quality alongside the water quantity targeted by the Water Sponge Challenge.
**(iv) Save Our Soils (S.O.S.) Challenge**	Halt soil loss through land-management practices and incentive structures that drive adoption at scale.	≥20% reduction in soil erosion through practices implemented by ≥ 40 growers or land managers across ≥ 3 managed land systems. Within 24 months.	**Intervention**	Provides a route to halt or reduce erosion across a diversity of growers and land systems, where adoption—not agronomic knowledge—is the rate-limiter.
**(v) Biological Fertilisers Challenge**	Reduce synthetic fertiliser use and associated N₂O emissions by deploying microbial inoculants and/or activating native nitrogen, phosphate and potassium remineralisation capacity.	≥25% reduction in synthetic fertiliser use and accompanying N₂O emissions, with no yield penalty, across 3 crop systems and 2 cropping seasons; demonstration in at least one of the three crops within 24 months as an interim milestone.	**Intervention**	Targets one of the largest cost, aquatic-pollution and emissions burdens in agriculture.
**(vi) Soil Health Diagnostic Test Challenge**	Develop a rapid, affordable assay that serves as an integrative measure of soil health—spanning physical, chemical and biological function—telling users whether soil is healthy, degrading or recovering.	Validated across ≥ 30 farms, ≥3 regions and ≥ 3 soil types; results within 1 week at low cost; predicts at least 4 of 5 core soil-health indicators (spanning physical, chemical and biological function) with >70% accuracy; applicable across major soil orders.	**Validation**	Creates a simple, trusted, and scalable way to assess whether soil is healthy, degrading or recovering after interventions, giving farmers, land managers, investors, and policymakers a shared basis for action.
**(vii) B.I.O.M.E. Challenge**	Develop a rapid, scalable approach to measure improvement in soil biocomplexity (the multitrophic microbial and faunal community) relative to a regionally defined context-appropriate reference condition.	Demonstrate ≥ 10% movement toward a regionally defined high-biocomplexity reference state using an existing biocomplexity metric. Within 24 months.	**Validation**	Captures ecological-integrity and biodiversity recovery in a way that is measurable, motivating, and globally applicable.

The integrated portfolio comprises five Intervention Challenges, which define actions to improve soil health, and two Validation Challenges, which establish how progress will be measured and compared. Winning thresholds are illustrative, provisional benchmarks intended for wider consultation and refinement. B.I.O.M.E., Biological Integrity, Observation, Monitoring and Evaluation; CO₂e, carbon dioxide equivalent; GHG, greenhouse gas; MRV, measurement, reporting and verification; N₂O, nitrous oxide.

This shortlist is intended as an integrated portfolio rather than independent ideas. Diagnostics, interventions, verification, deployment and community engagement reinforce one another and should ultimately be launched as a coordinated Heligan Grand Challenges platform.

### Day 3—public launch at Anthropy 2026, Eden Project

On 25 March, the meeting moved to the Eden Project, where the Soil Stars hosted two roundtables as part of the Anthropy 2026 forum. The morning session, “Soil—the quiet revolution,” was chaired by Sir Tim Smit with The Edge, Jo Handelsman, and Jack Gilbert, and was introduced by Lucy Harper on behalf of AMI. The midday session, “Save the soil. Soothe the soul. Share the solutions,” was chaired by Janet Jansson with Peter Byck, Tom Crowther, and Mark Stevenson. The two sessions introduced the soil crisis and the Heligan Grand Challenges to a public, business, and policy audience and framed them as a global call to action rather than a niche scientific output.

### Mission framing

As highlighted earlier, Jo Handelsman and Jack Gilbert led a series of group exercises in which the participants were asked to come up with X-Prize equivalent grand challenges that need to be solved in order to sustain the health of global soils. We worked to identify numerical targets through iterative expert deliberation, and each target was selected to be ambitious, measurable, and readily communicable. They represent provisional benchmarks rather than universal thresholds derived from formal modelling or systematic evidence synthesis and will be refined as their feasibility, baselines, and measurement protocols are evaluated by the wider community. The strongest concepts that emerged from the drafting day fall into two complementary categories:

- Intervention Challenges, which specify biological or biochemical solutions that aim to improve some facet of soil "health."
- Validation Challenges, which specify how those solutions will be evaluated, trusted, and compared. We describe the Intervention Challenges here and the Validation Challenges in the following section; the full portfolio is summarized in Table 1.

### Intervention challenges

**The Carbon Lock Challenge**, championed by participants working on the microbial drivers of soil organic matter stabilization, seeks biological pathways that drive plant- and microbially-derived soil carbon into durable, slow-turnover pools that are not sensitive to rapid mineralization. This is important as the majority of terrestrial carbon occurs in nonliving pools that can be either respired or not by microbes (Bar-On et al. 2025). The illustrative winning threshold is the identification of five carbon-lock molecules, and pathways to facilitate their increase, with measurable increases in the corresponding soil pools over 24 months, and credible evidence of stability within 36 months and predicted stability over decades to centuries. The challenge specifically links microbial ecology to climate mitigation, and to the measurement, reporting, and verification infrastructure that carbon markets will require if biological credits are to be implemented in a credible and trusted way.

**The Water Sponge Challenge** targets the rehydration of degraded and desiccated soils. Healthy soils retain moisture through the combined contributions of soil chemistry, physical structure, and a living biological community. Some microbes are particularly important for their ability to produce extracellular polysaccharides and proteins that glue soil particles together and reduce pore-water loss, and deep-rooted plant–microbial consortia simultaneously hold soil in place and pull moisture into deeper horizons. The challenge asks for either a 30% increase in soil water-holding capacity or a 20% reduction in irrigation demand in agricultural systems without yield or environmental penalty, validated across at least three soil types and climate regions within 24 months. The discussion drew explicitly on Paul Hawken’s point that the hydrological cycle is as central a climate variable as atmospheric carbon (Hawken 2017).

**The Water Purification Challenge** states that teams must reduce leaching of a target contaminant (nitrate, phosphate, or a degradable agrochemical) into streams and groundwater by ≥50% across at least three soil types and climate regions within 24 months. This could be achieved by restoring the soil biology that transforms or stabilizes the contaminant into a benign end-state, not by passive retention alone. To count, a reduction must satisfy three guardrails simultaneously. First, no pollution swapping, i.e. no net increase in soil greenhouse-gas emissions on a CO_2_-equivalent basis, with N₂O measured explicitly. Second, no soil-health penalty, i.e. maintenance or improvement of a defined soil-health panel (organic carbon, microbial biomass and activity, structure), with no accumulation of the parent contaminant or toxic metabolites; and third, demonstrated fate, i.e. a contaminant mass-balance showing where it went (mineralized, assimilated into biomass/stable organic matter, or stabilized in a nonleachable yet biologically compatible pool), so the reduction is a genuine transformation rather than temporary sorption that re-releases. The challenge connects soil recovery to water security and public health and complements the Water Sponge Challenge by addressing contaminant *quality* alongside water *quantity*.

**The Save Our Soils (S.O.S.) Challenge** moves from biological intervention into land management and incentive design. It asks for a 20% reduction in soil erosion, demonstrated by practices adopted across at least forty growers in three managed land systems within 24 months. The challenge acknowledges that the dominant causes of soil loss (including ploughing, annual crops with shallow root systems, lack of winter cover, and gully erosion) are well characterized, and that the rate-limiter is now adoption of practices rather than agronomic knowledge. The known solution set, including intercropping with deep-rooted perennials, winter cover crops, between-row living groundcover, reduced tillage, and AMP grazing of grasslands is incorporated into the challenges’ judging criteria.

**The Biological Fertilizers Challenge** is among the most operationally ambitious that was proposed. It asks teams to demonstrate a 25% reduction in synthetic fertilizer use and accompanying nitrous oxide emissions, across three crop systems and two cropping seasons, with no yield penalty. This could be achieved through microbial inoculants or activation of native nitrogen-, phosphate-, and potassium-remineralization capacity. The drafting team explicitly noted that this challenge crosses several of the regulatory frontiers identified in the cross-cutting enablers section below, and that demonstrating that the intervention works in at least one of the three target crops within 24 months is a deliberately lower-friction interim milestone designed to keep this challenge open to a wide field of solvers.

### Validation challenges

Diagnostics are the silent infrastructure of soil-health innovation. Without rapid, affordable, trusted tools to determine whether a soil is healthy, degrading, or recovering, even the most elegant biological intervention will struggle to attract investment, regulatory approval, or farmer uptake. Two Validation Challenges were therefore drafted alongside the five Intervention Challenges and given equal weight in the portfolio.

**The Soil Health Diagnostic Test Challenge** (referred to often by participants as a “soil blood test”) asks innovators to develop a rapid, affordable assay (results available within one week at low cost), validated across at least thirty farms, three regions and three soil types. The assay must serve as an integrative measure of soil health rather than any single property; therefore it must predict at least four of five core soil-health indicators spanning the physical, chemical and biological dimensions of function, each with greater than 70% accuracy, and remain applicable across major soil orders. The candidate indicator set includes biological activity and microbial functional capacity (e.g. respiration, enzyme activity, microbial biomass), carbon and organic-matter dynamics, including soil organic matter accumulation/stabilization potential, nutrient cycling and availability (e.g. N and P supply capacity), soil structure and water function (aggregate stability, infiltration, water-holding capacity), and resilience/buffering (e.g. disease suppressiveness or contaminant attenuation). The drafting discussion centered on the trade-off between assay simplicity (necessary for global affordability and adoption) and biological information density (necessary for the diagnostic to genuinely reflect soil microbial function); the preferred direction was a hybrid of metabolomic and microbial functional markers, interpreted through AI-assisted decision-support tools allowing region- and crop-specific calibration.

**The B.I.O.M.E. (Biological Integrity, Observation, Monitoring and Evaluation) Challenge** addresses the biological complexity of soils directly. It asks teams to demonstrate at least a 10% movement toward a regionally defined high-biocomplexity reference state within 24 months. Whereas the Soil Health Diagnostic predicts agronomic and ecological function, BIOME evaluates ecological integrity as a distinct dimension of soil recovery by comparing a soil’s multitrophic community with a context-specific reference condition. Such references may include minimally disturbed sites where appropriate, but the Challenge does not assume that unmanaged natural communities or maximal biodiversity are optimal for managed soils. How regional, soil and land-use contexts should inform reference selection remains to be developed and validated through wider consultation. The premise is a robust, region-specific biocomplexity baseline against which recovery can be scored. Rather than inventing new metrics, BIOME adapts established “distance-to-reference” frameworks to the soil subsurface: holistic, multiscale biocomplexity indices that already include a microbial dimension (the SEED Biocomplexity Index (McElderry et al. 2023)), abundance- and integrity-based measures benchmarked to undisturbed sites, e.g. the Biodiversity Intactness Index (Scholes and Biggs 2005) and Ecosystem Integrity Index (Hill et al. 2022), and soil-native community metrics that quantify food-web complexity and faunal diversity directly, e.g. nematode faunal-analysis indices (Ferris et al. 2001) and the QBS-ar soil biological quality index (Parisi et al. 2005). Combined, these provide microbial-through-faunal coverage and a 0–1 recovery scale suitable for region- and soil-order-specific calibration.

### Cross-cutting enablers

The portfolio of grand challenges rests on four cross-cutting enablers that any successful deployment must address. These were repeatedly identified across the drafting day, and are summarised graphically in Fig. 1.

***Enabler 1. Validation infrastructure*.** Without harmonized monitoring, common benchmarks, and independent auditing, claims made in one geography or production system cannot be evaluated credibly in another. The European Union’s Mission: A Soil Deal for Europe is the most mature example of such an infrastructure, with 100 living labs and lighthouses targeted by 2027 and the EU Soil Observatory’s nineteen-indicator dashboard providing harmonized metrics for national scale monitoring. Bridget Emmett’s contribution made clear that this is a model the Heligan Grand Challenges can align with rather than duplicate, particularly for the Validation Challenges although a clear set of benchmark sites could help both initiatives.

***Enabler 2. Scalability***. Microbial inoculants and other biologically based products must not only work biologically; they must be manufacturable, storable, distributable, affordable and robust to local environmental conditions and resident microbiota. Participants emphasized formulation, delivery, affordability, farmer co-design and regional adaptation as integral to the Challenges rather than downstream considerations. They also recognized that greenhouse and small-plot efficacy is insufficient to establish utility: candidate interventions ultimately require multilocation, multiseason evaluation under commercial farming conditions, including agronomic performance, operational feasibility and economic value.

***Enabler 3. Regulation and biosafety*.** Microbial products do not fit neatly into policy frameworks originally designed for chemical fertilizers, conventional pesticides, or genetically modified plants. The Organization for Economic Co-operation and Development (OECD) has long recognized the need for dedicated environmental safety evaluation frameworks for microbial biocontrol agents (OECD 2014), and Eckerstorfer and colleagues have recently argued that microorganisms may require a distinct environmental risk-assessment approach because their persistence, dispersal, reproduction, and ecological interactions after release are difficult to predict (Eckerstorfer et al. 2025). A recently proposed risk-proportional, release-based stewardship framework provides a practical operational approach by combining strain-resolved identification and hazard screening with ecological testing, monitored field deployment, and a federated registry for traceability and adaptive governance (Edlund et al. 2026). The draft Heligan Challenges will draw directly on this framework with regulatory readiness, traceability, environmental risk assessment, and post-release stewardship built into the judging criteria from the outset rather than treated as downstream considerations.

***Enabler 4. Economics, policy alignment, and public legitimacy***. Farmers face strong incentives to maintain the status quo because the costs of transition to regenerative practice are immediate and the benefits of soil restoration accrue slowly and often outside the farm gate. Reviews of regenerative and sustainable agriculture identify recurring barriers to adoption, including upfront financial pressure, uncertain short-term returns, inconsistent metrics, limited extension support, lack of insurance backing for transition, and institutional barriers in supply chains and policy systems (Kroh et al. 2026). Anna Edlund made a particular case for treating the financial architecture as a leverage point; e.g. crop insurance in most markets are structured around recent yield outcomes, misaligning incentives with soil health investments whose benefits accrue over years to decades, and the insurance and banking sectors are underused partners in designing incentive structures for regenerative agriculture. Jane Franch and Peter Byck contributed contextual examples of how US Department of Agriculture incentive structures have, in their view, historically reinforced rather than challenged the dominant chemical-input model, and how Brazilian biological-nitrogen programmes provide a contrasting demonstration of what is possible when regulatory and financial systems align.

***Enabler 5. Engaging public narrative*.** A recurring theme of the meeting was that progress requires both stronger evidence for consistent field performance and more effective engagement with farmers, policymakers and the public. Narrative, attention, and trust were therefore identified as deployment barriers alongside continuing scientific uncertainties. The Heligan Challenges were therefore designed not only as research targets but as communicable stories with measurable thresholds. The grand challenge names and the deliberate XPRIZE framing are part of that design. The Day 2 evening conversation specifically considered how to bring more musicians, filmmakers, artists and civic actors into the Soil Stars network, and the Day 3 Anthropy launch was structured as a public-facing demonstration that scientific and cultural voices can credibly share a stage on this issue.

### Outcomes and next steps

The immediate outcome of the meeting is the first draft of the Heligan Protocols, including the seven Intervention and Validation grand mission challenges (Table 1, Fig. 1). The drafting represents the start, not the end, of the work; the Protocols continue to evolve through Soil Stars working groups, and several outputs are already underway.

First, a formal Heligan Challenges framework paper is in preparation. The intent is a peer-reviewed publication that provides a full community-approved justification of the portfolio, explains the logic of the Intervention/Validation split, and sets out detailed principles for validation, ethics and deployment. Second, a public-facing platform at The Soil Stars (thesoilstars.com) will present each challenge as a mission page with a public narrative, performance criteria, timelines and partner opportunities, designed for use by farmers, investors, regulators, and the general public as well as by the research community. The Challenges and associated outputs will be publicly available and company-neutral; participation confers no endorsement, preferential access, intellectual-property or data rights, or exclusive commercial benefit. Third, the Soil Stars network is working toward a coordinated funding ambition that combines philanthropic, public, and private sources to enable the grand challenge prizes themselves and the underlying research infrastructure. Fourth, groups in Brazil, the United States, and several African nations are working to establish real-world demonstration networks aligned with the Challenges. Fifth, the summit identified the IUCN Microbial Conservation Specialist Group, launched in 2025 (Gilbert et al. 2025), as a natural sister venture whereby the MCSG addresses microbes as targets of conservation, whereas the Heligan Challenges address microbes as engines of recovery, and the two share many of the same enablers.

We end with the observation that has run through every Soil Stars meeting and every external response to this one, that soil recovery is no longer a scientific aspiration but a deployable agenda, and that what remains is a coordinated, mission-led architecture in which solvers can converge. The Heligan Challenges are a first attempt to build that architecture. The missions, challenges and protocols presented here are preliminary outputs of the Heligan Summit rather than finalized community standards. They provide a starting point for broader international and cross-sector consultation involving researchers, farmers, industry, policymakers and other stakeholders. The next phase will seek broader participation through the Soil Stars and partner scientific networks. Opportunities to provide feedback and contribute to the continued development of individual challenges and protocols will be publicized through the Soil Stars website, community publications, and presentations and discussion sessions at scientific meetings.

## Data Availability

No data resources are presented or used in this paper.
